# Electromagnetic field redistribution induced selective plasmon driven surface catalysis in metal nanowire-film systems

**DOI:** 10.1038/srep17223

**Published:** 2015-11-25

**Authors:** Liang Pan, Yingzhou Huang, Yanna Yang, Wen Xiong, Guo Chen, Xun Su, Hua Wei, Shuxia Wang, Weijia Wen

**Affiliations:** 1 Soft Matter and Interdisciplinary Research Center, College of Physics, Chongqing University, Chongqing, 400044, P. R. China; 2 Department of Applied Physics, College of Physics, Chongqing University, Chongqing, 400044, P.R. China; 3 Department of Physics, The Hong Kong University of Science and Technology, Clear Water Bay, Kowloon, Hong Kong, China

## Abstract

For the novel interpretation of Raman spectrum from molecule at metal surface, the plasmon driven surface catalysis (PDSC) reactions have become an interesting topic in the research field of surface enhanced Raman scattering (SERS). In this work, the selective PDSC reactions of p,p’-dimercaptoazobenzene (DMAB) produced from para-aminothiophenol (PATP) or 4-nitrobenzenethiol (4NBT) were demonstrated in the Ag nanowires dimer-Au film systems. The different SERS spectra collected at individual part and adjacent part of the same nanowire-film system pointed out the importance of the electromagnetic field redistribution induced by image charge on film in this selective surface catalysis, which was confirmed by the simulated electromagnetic simulated electro- magnetic field distributions. Our result indicated this electromagnetic field redistribution induced selective surface catalysis was largely affected by the polarization and wavelength of incident light but slightly by the difference in diameters between two nanowires. Our work provides a further understanding of PDSC reaction in metal nanostructure and could be a deep support for the researches on surface catalysis and surface analysis.

Due to the charming properties of manipulating light under sub-wavelength, plasmon experiences a rapid expansion since the beginning of this century and as an emerging interdiscipline attracts tremendous interesting from scientific researchers not only in physics, but also chemistry, biology, materialogy, etc^1^,^2^,^3^. This fantastic ability of bounding light originate from the collective oscillation of free electrons near metal surface excited by light, which is called surface plasmon polaritons (SPPs). The generation of SPPs in metal nanostructures results in the nonuniform distribution of electromagnetic fields near metal surface. When the resonance oscillation of free electrons occurs that called localized surface plasmon resonance (LSPR), this nonuniform reaches maximal and makes electromagnetic field in some areas near metal surface obtain extremely enhanced. This hugely enhanced electromagnetic field brings about a dramatically improvement in efficiency of various optical processes on surfaces, such as surface enhanced Raman scattering (SERS)^4^,^5^,^6^, plasmonic driven surface catalysis (PDSC)^7^,^8^,^9^,^10^, hot electron generation^11^,^12^, second-harmonic effects^13^,^14^, plasmonic trapping^15^,^16^, plasmonic enhanced optical activity^9^,^17^,^18^, plasmonic sensor^19^,^20^,^21^, etc.

Among these plasmonic enhanced optical phenomena, the researches on PDSC are of important theoretical significance and application prospects. In the last twenty years, the three additional Raman peaks of PATP adsorbed on metal surface at 1143, 1390 and 1432 cm^−1^ was commonly thought as the results of chemical enhancement in SERS. However, in 2009 researchers theoretically predicated these three Raman came from the new molecule DMAB but not PATP that there was a photocatalysis progress at metal surface called PDSC^22^, which was soon demonstrated in the same year at the surface of Ag film and Ag nanospheres^8^,^23^. In the following five years, the researches in this area have explored rapidly that different metal (Au, Ag, Cu) nanostructures such as nanowire^24^, nanospheres^25^, nanoparticle-film^26^ have been investigated and another PDSC reaction of DMAB generated by 4NBT was also reported^7^. To explore the feature of molecules and metal surface in PDSC reaction is the key mission of this scientific field which is quite important for distinguishing SERS signals from origin targets or the new catalyzed molecule.

Since this surface catalysis process requires SPPs with high energy to break the chemical bond (N-H in PATP and N-O in 4NBT), the status of SPPs near the molecule is quite important to the PDSC reactions. As one known, the structure factor of metal surface dominates the properties of SPPs^1^,^27^. Therefore, to verify the PDSC process in various metal nanostructures is the crucial issue in this area. Because of simple construction and convenient fabrication, metal nanoparticle-film systems are the widely used SERS substrates in chemical and biological sensing^20^,^28^. In recent two years, the electromagnetic field redistribution caused by the coupling of surface charge on nanoparticle and image charge on film in the systems has been reported that the confined light energy could be modulated at different sub-wavelength areas through manipulating wavelength of incident light^29^,^30^,^31^. Therefore, studying the influence of the electromagnetic field redistribution on PDSC reactions is the interesting frontier researches in related fields.

In previous reports on electromagnetic field redistribution, the nanoparticle monomer and dimer consisted of metal nanoparticle with similar sizes and shapes were chosen to investigate the coupling between surface charge on particle and image charge on film^30^,^31^. However, due to the intrinsic restriction of nanofabrication technology, it is very hard to find two nanoparticles with the exactly same sizes, shapes and distance of nanoparticle-film gap. As one know, the tiny structural variation of metal surface, especially for the gaps between nanostructures, results in the great difference in SPPs coupling^27^. Therefore, the minute structural difference of nanoparticles with similar sizes and shapes is the troublesome problem in the strict demonstration of electromagnetic field redistribution.

Since the vertical oscillation of SPPs in nanowire could be seen as that in nanoparticle with size equal to the diameter of nanowire, the minute structural difference of nanoparticle is overcome by the two adjacent nanowires on film. When the polarization of incident light is perpendicular to the direction of nanowires, the individual part of nanowires (the two terminals of the Ag nanowire dimer, illustrated by the blue arrow in Fig. 1(a)) could be treated as the nanoparticle monomer and the adjacent part of nanowires (the middle of the Ag nanowire dimer, illustrated by the blue arrow in Fig. 1(b)) could be treated as the nanoparticle dimer. Because of the uniform diameter of nanowire and the same nanowire-film gap, the individual nanowire and nanowire dimer become the perfect system to verify the electromagnetic field redistribution. In this work, the PDSC reaction of DMAB produced by PATP and 4NBT was demonstrated in the Ag nanowires-Au film systems. In this metal nanowire-film system, the SERS spectra from nanowire dimer-film gaps exhibited the obvious Raman feature of DMAB while that from individual nanowire-film gaps presented the behavior of PATP or 4NBT.

These experimental results not only demonstrated strictly the interesting theoretical predication of electromagnetic field redistribution but also indicated its great influence on the PDSC reactions. The theoretical and experimental results also figured out the importance of incident polarization and nanowire diameter in the PDSC reactions. Our studying expanded the understanding of PDSC reactions and had great significance in field of SERS and surface analysis.

## Results

### SERS spectra of 4NBT adsorbed on Au film at different positions of Ag nanowires with similar diameters excited by 633 nm laser

**Figure 1:**
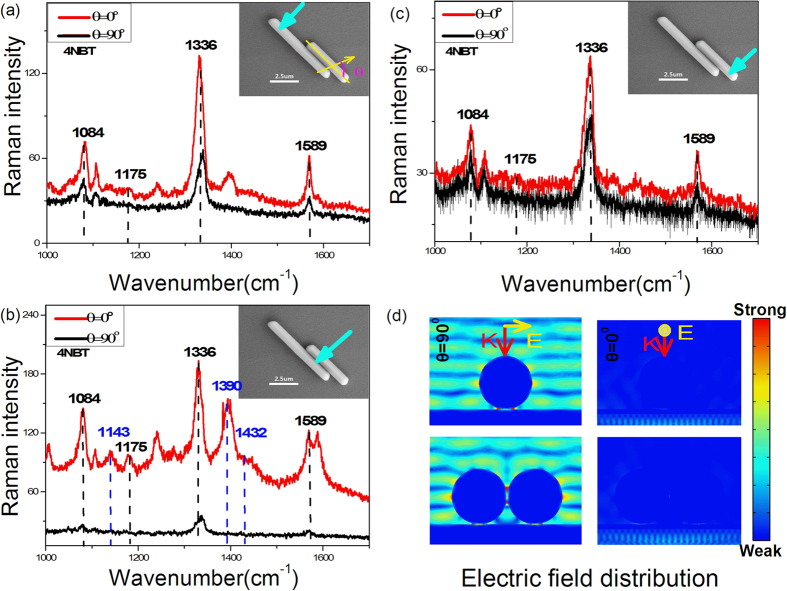
SERS spectra of 4NBT adsorbed on Au film and distribution of electric field at different positions of Ag nanowires with similar diameters excited by 633 nm laser. (**a**) The left individual point of the nanowires(as indicated by the blue arrow), (**b**) the adjacent point of the nanowires, (**c**) the right individual point of the nanowires, (**d**) E field distribution in the top individual point and the adjacent point. Insets are the corresponding SEM image of Ag wire-Au film system.

A monolayer of 4NBT was adsorbed on a 100 nm thick Au film evaporated on the Si substrate. Then the Ag nanowires were randomly located on the Au film with molecule monolayer between them through spin coating method. Using a mirco-Raman spectrometer, the 633 nm laser excited SERS spectra of 4NBT adsorbed on Au film were collected at different positions of two parallel adjacent Ag nanowires with similar diameters as shown in Fig. 1. The inset SEM images of Ag nanowires figured out the diameters were both about 920 nm while the lengths were 7.5 μm (left) and 4.9 μm (right) with a 3.5 μm adjacent part. As the blue arrow illustrated in SEM image, SERS signals with different polarization of incident light were firstly collected at the individual part of left nanowire, which were shown in Fig. 1(a). Both the parallel excited (θ** = 0**^**o**^, black line) and the perpendicular excited (θ** = 90**^**o**^, red line) spectra presented the obvious Raman feature of 4NBT (Raman peaks at 1084 cm^−1^, 1175 cm^−1^, 1336 cm^−1^, 1589 cm^−1^), although a larger Raman intensity appeared in the perpendicular excited one. Where the θ is the angle between the polarization direction of the incident light to the axis of the silver nanowires. However, the results changed a lot when the position of collected SERS moved to the adjacent part of nanowires as illustrated in Fig. 1(b). First, the intensity difference between two polarizations was much larger that it was almost 8 times greater for Raman intensity of perpendicular excited compared to parallel excited at 1084 cm^−1^ Raman peak. Second, more Raman peaks appeared in the perpendicular excited situation while there were only three Raman peaks at 1084 cm^−1^, 1175 cm^−1^, 1336 cm^−1^, 1589 cm^−1^ presented in parallel excited spectrum. Furthermore, the additional Raman peaks at 1143 cm^−1^, 1390 cm^−1^, 1432 cm^−1^ in perpendicular excited spectrum confirmed the generation of DMAB produced by 4NBT through PDSC reactions. Interesting, the results came back when the collected position moved to the individual part of the right nanowire as shown in Fig. 1(c). Although there were only Raman peaks of 4NBT such as 1084 cm^−1^, 1175 cm^−1^, 1336 m^−1^, 1589 cm^−1^ exhibited in both perpendicular and parallel excited spectra, the perpendicular one still had a little stronger Raman intensity. To understand the variation of SERS spectra in the nanowire system, the distribution of near fields were also investigated through COMSOL method here. In Fig. 1(d), the left two images illustrated the enhanced electromagnetic distribution of individual part and adjacent part of nanowire in perpendicular excited situation, in which the yellow and red arrows represented the polarization and propagation directions of incident light respectively. It was obvious that the electric field intensity in nanowire-film gap of adjacent part was much stronger than that of individual part. Because the SERS intensity is proportion to (E/E0)^4, this simulation result presented a good explanation of much greater Raman intensity obtained at adjacent part in Fig. 1(b). In the left two images in Fig. 1(d), the monolayer of 4NBT (or PATP) molecule is only adsorbed on the Au film. The great enhance electric field found in the nanowire-nanowire gap had no contribution to the SERS intensity. At the meantime, the right two images in Fig. 1(d) indicated the enhanced electric field of the nanowire system in parallel excited situation. Obviously, the electric field intensity here was much weaker compared to the perpendicular excited one that it was too weak to distinguish the redistribution of electric field between individual part and adjacent part. This simulation results were consistent with our SERS experiment results that all the Raman intensities in parallel excited spectra collected at different position of this nanowire-film system was weaker.

### SERS spectra of 4NBT adsorbed on Au film at different positions of Ag nanowires excited by 532 nm (~2.8 mW) and 633 nm (~1.7 mW) laser

**Figure 2:**
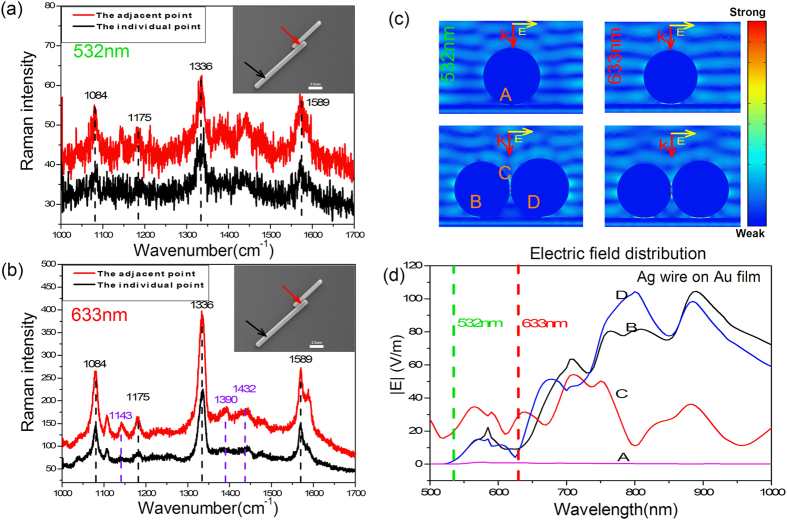
SERS spectra of 4NBT adsorbed on Au film and distribution of electric field at different positions of Ag nanowires perpendicular excited by 532 nm and 633 nm laser. (**a**) When excited by 532 nm, (**b**) When excited by 633 nm, (**c**) E field distribution of the points shown in insets (red and black arrow in (**b**)), (**d**) E field enhancement in gaps A, B, C, D (shown in (**c**)).

To understand the wavelength influence on PDSC in this nanowire-film system, SERS spectra of 4NBT adsorbed on Au film illuminated by 532 nm or 633 nm laser were investigated at the different positions in the same system. Considering the plasmon coupling between nanowires and also between nanowire and film in parallel excited situation was too weak to investigate the redistribution of electromagnetic field, only the perpendicular excited results were discussed in the following sections. The inset SEM image in Fig. 2 indicated the two Ag nanowires figured out the lengths were 6.9 μm and 12.2 μm with adjacent part was 2.3 μm. Although the intensities of 532 nm laser excited SERS spectra in Fig. 2(a) were weak, it was still obvious to distinguish the Raman feature of 4NBT such as peak at 1084 cm^−1^, 1175 cm^−1^, 1336 cm^−1^, 1589 cm^−1^ in both spectra from the individual part (back line) and adjacent part (red line), whose collected positions were indicated by black and red arrows in SEM image respectively. Meanwhile, the SERS spectra excited by 633 nm laser in the same nanowire-film system in Fig. 2(b) presented much different results here. First, the Raman intensities were much larger (~10 times) than these excited by 532 nm laser at 1084 cm^−1^ Raman peak. Second, the more Raman peaks such as 1143 cm^−1^, 1390 cm^−1^, 1432 cm^−1^ in the spectrum from adjacent part (red line) figured out the generation of DMAB while the spectrum from individual part (black line) only exhibited the Raman feature of 4NBT. To understand this phenomenon, the corresponding near field distribution at 532 nm or 633 nm in this nanowire-film system was studied and the simulation results was illustrated in Fig. 2(c), where the nanowire-film gap at individual part, the nanowire-film gap at adjacent part (left and right) and the nanowire-nanowire gap were defined as gap A, gap B, gap D and gap C. It was obvious that the electric field intensities in gap B and D much greater than that in gap A, which was consistent with our SERS spectra no matter the wavelength of incident laser was 532 nm or 633 nm. Furthermore, the weaker electric field intensity at 532 nm in the same system gave contribution to the stronger SERS intensity at 633 nm, which was also partly ascribed to the 3d electron transition of Au material at 532 nm. The detail wavelength dependence of enhanced electric field distribution was illustrated in Fig. 2(d) where the green and red dotted lines represented the 532 nm and 633 nm. The variation of electric field intensities in various gaps here indicated the confined area of light energy by SPPs was greatly influenced by wavelength. In the whole spectra, the electric field intensities in nanowire-film gap B (black line) and D (blue line) were almost the same which were always higher than that in gap A (pink line). This meant the adjacent part in system had greater ability to confine light on film than the individual part. However, it was more complex in adjacent part as one focused on the electric field intensity in gap C (red line). When the wavelength was smaller than ~645 nm, the electric field intensity in nanowire-nanowire gap C was greater than that in nanowire-film gaps (either gap B or D). This meant the SPPs preferred to confine light into nanowires but not on film, which was confirmed by the images of electric field distribution in Fig. 2(c). Interesting, when the wavelength was larger than ~645 nm, the much faster increased blue and black line compared to red line in Fig. 2(d) indicated the nanowire-film system had more powerful ability to confine light on film at larger wavelength. Here the difference of electric field intensities in gap B and D was contributed to the minute difference in diameter of two nanowires which were 1012 nm and 1070 nm respectively. In our experiment (532 nm or 633 nm), the strongest field enhancement occurs in gap between neighboring nanowires (gap C). However, in this work the PATP or 4NBT molecule is only adsorbed on Au film but not Ag nanowires, whose PDSC reactions were dominated by the surface plasmon in gap between Ag nanowire and Au films (gap B or D). Therefore, although the great enhanced electric field generated in the gap between neighboring nanowires, it had no contribution to the SERS intensity.

### SERS spectra of 4NBT and PATP adsorbed on Au film at different positions of Ag nanowires with different diameters

**Figure 3:**
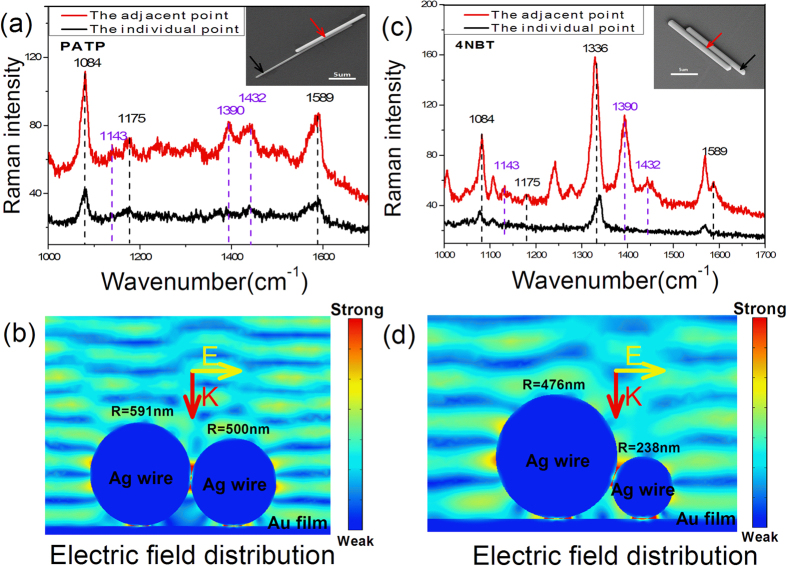
SERS spectra of 4NBT and PATP adsorbed on Au film and distribution of electric field at different positions of Ag nanowires with different diameters. (**a**) SERS spectra of 4NBT, (**b**) E field distribution of the Ag wires-Au film (shown in the inset of picture (**a**)), (**c**) SERS spectra of PATP, (**d**) E field distribution of the Ag wires-Au film (shown in the inset of picture (**c**)).

Since the diameter of Ag nanowire had an effect on the intensity of enhanced electric field in nanowire-film gaps as described above, here the two adjacent Ag nanowires with different diameters were also investigated in this nanowire-film system. As the SEM image inset in Fig. 3(a), two Ag nanowires with a 10.7 μm micro adjacent part were located on the 4NBT monolayer adsorbed on Au film, where the diameters of two nanowires were 1182 nm and 1000 nm and the length of them were 14 μm and 13.5 μm respectively. The two SERS spectra excited by 633 nm in Fig. 3(a) were collected at individual part (black line) and adjacent part (red line) as the arrows indicated. Although the diameters was different, the phenomenon of SERS spectra were almost the same that the much stronger Raman feature of DMAB were exhibited in the SERS spectra from adjacent part while that from individual part only present the weaker Raman feature of 4NBT. The corresponding electric field distribution at 633 nm in Fig. 3(b) were further confirmed the SERS conclusions that the nanowire-film gaps had greater electric field intensity at adjacent part than that at individual part. Furthermore, another two adjacent Ag nanowires with great difference in diameters were also studied in this work. The SEM image inset in Fig. 3(c) illustrated these two nanowires with a 15.4 μm adjacent part whose length were 19.8 μm and 27.8 μm with the diameter were 952 nm and 476 nm. In this nanowire-film system, the SERS spectra had also exhibited the selective catalysis of position that the Raman feature of DMAB was presented at adjacent part but not at individual part, which was further confirmed by the electric field distribution in Fig. 3(d). Since the two adjacent Ag nanowires in Fig. 3(c,d) were located at the PATP monolayer adsorbed on Au film, this selective catalysis in nanowire-film system was compatible to either 4NBT or PATP.

## Discussion

### Through SERS of 4NBT or PATP monolayer adsorbed on Au film, the electromagnetic field redistribution is demonstrated in two adjacent nanowires located on film to exclude the difference in size and shape between nanoparticles

The polarization dependence of SERS intensities at the same position exhibited in Fig. 1 could be understood by the excitation and coupling of SPPs. When polarization of incident light is parallel to the nanowires, the oscillation of SPPs are along the axis of nanowire and the coupling of SPPs between nanowires is so weak that the adjacent part of two nanowires could be treated as two individual nanowire. But in the perpendicular excited situations, the oscillation of SPPs are vertical to the axis of nanowires and a strong plasmon coupling occurred between two adjacent nanowires that it could be treated as two adjacent nanoparticles. Considering the uniform of diameter in chemical synthesized Ag nanowire, the individual part and adjacent part of nanowire could be seen as the two nanoparticles in monomer or dimer with the same shapes and size in perpendicular excited situations. The 532 nm or 633 nm excited SERS spectra collected at individual or adjacent part in Fig. 2(a,b) demonstrated the selective catalysis in this nanowire-film system, which was consist with the corresponding simulated electric field distribution in Fig. 2(d). As indicated by green and red dash line, the electric field intensities in nano-film gap at adjacent part (gap B and D) were always lager than that at individual part (gap A), which were almost 8 time’s greater at 532 nm and 11time’s greater at 633 nm. This enhanced electric field could be understood by the electromagnetic field redistribution. With the help of induced image charge on Au films, the effective electric dipoles were located at the nanowire-film gaps at the adjacent part but not the individual part, which was similar to our previous work in the nanoparticle dimer system^25^,^26^,^30^,^31^. Considering the SERS intensity is proportion to (E/E0)^4, this field enhancement could bring out great difference of SERS intensity in different position at Ag nanowire-Au film system. However, the SERS intensities in corresponding experiment measurement were not so much larger at the adjacent part in Fig. 2(a,b), which was almost 2 times larger at 633 nm (or 532 nm). Besides 3d electron transition of Au material at 532 nm and the experiment error (such as the accurate position of Ag nanowire could not be controlled exactly at the center of focused light with 2–3 μm diameter because of diffraction limit), this difference in experiment and simulation may be attributing to the plasmon wavegude on Ag nanwire. In our SERS experiment, the diameter of incident light focused by an objective was about 2–3 μm while the lengths of the adjacent part in Fig. 2 were 2.3 μm, which means the terminals of the Ag nanowire was probably illuminated by incident light. According to previous reports on plasmon waveguide^32^,^33^, the light could couple into propagating SPPs on Ag nanowire at the defect (such as terminals, adjacent nanoparticle) to generate plasmon waveguide. In the SERS measurement of Ag nanowire dimer in Fig. 2, the light could be lost by waveguide that the weaker enhancement at adjacent part was obtained. This reason could also be responsible for the not so much larger SERS intensities at adjacent part in Fig. 1, in which the length of adjacent part was just 3.5 μm. Whatever, the three DMAB Raman peak exhibited in both Figs 1 and 2 demonstrates the greater field enhancement at adjacent part compared to individual part. In our supporting information, the Ag nanowire dimer with longer adjacent part (about 5.6 μm) was investigated at the similar experiment environment. The collected Raman signals exhibited much larger intensities that it was almost 11 time’s greater at 1336 cm^−1^ Raman peak and 14 time’s greater at 1589 cm^−1^ Raman peak. The transfer of electromagnetic energy confinement from nanowire-nanowire gap to nanowire-film gaps manipulated by wavelength in Fig. 2(d) could be understood by the fact that the excitation and coupling of SPPs are greatly influenced by the wavelength of incident light, which is consistent with the reports in nanoparticle-film system^25^,^26^,^30^,^31^. In this work, the diameter of nanowire is found to have an influence on the electromagnetic field redistribution although it didn’t overturn the conclusion that the adjacent part has stronger ability to confine light on film. Since there is always difference in size or shape between nanoparticles, the diameter influence is interesting to be further studied.

In summary, the PDSC reactions of DMAB converted from 4NBT and PATP were investigated in the metal nanowire-film system. Thanks to the uniform diameter of the same nanowire, the influence of size difference in nanoparticle dimer on electromagnetic field redistribution is excluded in this nanowire-film system. The results indicated the PDSC reaction is performed at adjacent part of two nanowires for much larger electric field produced by induced image charge on film. This electromagnetic field redistribution induced selective surface catalysis is largely affected by the wavelength of incident light but slightly by the difference in diameters between two nanowires. Owing to the simple structure and convenient operation of nanowires-film systems, the electromagnetic field redistribution induced surface catalysis in our works is applied widely not only in chemical catalysis on metal surface, but also in other plasmonic fields such as environment sensor, photon detection, water splitting, and etc.

## Methods

PATP and 4NBT were all purchased from Aladdin Industrial Corporation.

Under the condition of high vacuum, electron beam evaporation system (model Peva-600E) was used to evaporate Au layer (100 nm thick) on silicon as the substrate for SERS measurement. By the atomic force microscopy (AFM) images, the average surface roughness of Au film was evaluated to be 2.249 nm

After centrifugal washing for several times, Ag nanowire in ethanol solution with low concentration is dropped on Au film through spin coating method. Because of the surface tension in the rapid evaporation of ethanol, some Ag nanowires are gathered to generate Ag nanowire dimer. Since this gathering is not tight enough, the gap distance between nanowires is estimated to be 1 nm. And the layer thickness between Ag nanowires and Au film is also estimated to be 1 nm for the PATP or 4NBT molecule monolayer is adsorbed on Au film.

Using a commercial Micro-Raman spectrometer (Horiba) with a 532 nm laser matched 5% filter or 633 nm matched 10% filter, their total output power is 57mW and 17mW respectively, So when the laser is irradiated to a sample though filter, their intensity is nearly 2.8mW and 1.7mW.

The electromagnetic field redistribution of the metal nanowire-film systems was simulated using the finite element method (COMSOL 4.3b commercial package). The nanowire located 1 nm above the Au films (100 nm thick) evaporated on the Si substrate. The metal nanowire-film systems used in this paper have two nanowires which parallel adjacent to each other with 1 nm edge-to-edge dimer seperation.

## Additional Information

**How to cite this article**: Pan, L. *et al.* Electromagnetic field redistribution induced selective plasmon driven surface catalysis in metal nanowire-film systems. *Sci. Rep.* **5**, 17223; doi: 10.1038/srep17223 (2015).

## Supplementary Material

Supplementary Information

## References

[b1] Giannini V., Fernandez-Dominguez A. I., Heck S. C. & Maier S. A. Plasmonic Nanoantennas: Fundamentals and Their Use in Controlling the Radiative Properties of Nanoemitters. Chem Rev 111, 3888–3912; 10.1021/Cr1002672 (2011). 21434605

[b2] Ozbay E. Plasmonics: Merging photonics and electronics at nanoscale dimensions. Science 311, 189–193; 10.1126/science.1114849 (2006). 16410515

[b3] Barnes W. L., Dereux A. & Ebbesen T. W. Surface plasmon subwavelength optics. Nature 424, 824–830; 10.1038/nature01937 (2003). 12917696

[b4] Li J. F. *et al.* Shell-isolated nanoparticle-enhanced Raman spectroscopy. Nature 464, 392–395; 10.1038/Nature08907 (2010). 20237566

[b5] Xu H. X., Bjerneld E. J., Kall M. & Borjesson L. Spectroscopy of single hemoglobin molecules by surface enhanced Raman scattering. Phys Rev Lett 83, 4357–4360; 10.1103/PhysRevLett.83.4357 (1999).

[b6] Huang Y., Fang Y., Zhang Z., Zhu L. & Sun M. Nanowire-supported plasmonic waveguide for remote excitation of surface-enhanced Raman scattering. Light: Science & Applications 3, e199; 10.1038/lsa.2014.80 (2014).

[b7] Sun M. & Xu H. A novel application of plasmonics: plasmon-driven surface-catalyzed reactions. Small 8, 2777–2786; 10.1002/smll.201200572 (2012). 22777813

[b8] Huang Y. F. *et al.* When the Signal Is Not from the Original Molecule To Be Detected: Chemical Transformation of para-Aminothiophenol on Ag during the SERS Measurement. J Am Chem Soc 132, 9244–9246; 10.1021/Ja101107z (2010). 20527877

[b9] Sun M. *et al.* Remotely excited Raman optical activity using chiral plasmon propagation in Ag nanowires. Light: Science & Applications 2, e112; 10.1038/lsa.2013.68 (2013).

[b10] Sun M., Zhang Z., Kim Z. H., Zheng H. & Xu H. Plasmonic scissors for molecular design. Chemistry 19, 14958–14962; 10.1002/chem.201302610 (2013). 24038434

[b11] Mubeen S. *et al.* An autonomous photosynthetic device in which all charge carriers derive from surface plasmons. Nat Nanotechnol 8, 247–251; 10.1038/nnano.2013.18 (2013). 23435280

[b12] Knight M. W., Sobhani H., Nordlander P. & Halas N. J. Photodetection with Active Optical Antennas. Science 332, 702–704; 10.1126/science.1203056 (2011). 21551059

[b13] Metzger B. *et al.* Doubling the Efficiency of Third Harmonic Generation by Positioning ITO Nanocrystals into the Hot-Spot of Plasmonic Gap-Antennas. Nano Lett 14, 2867–2872; 10.1021/nl500913t (2014). 24730433

[b14] Aouani H. *et al.* Multiresonant Broadband Optical Antennas As Efficient Tunable Nanosources of Second Harmonic Light. Nano Lett 12, 4997–5002; 10.1021/Nl302665m (2012). 22916834

[b15] Li Z. *et al.* Ultrasensitive size-selection of plasmonic nanoparticles by Fano interference optical force. ACS nano 8, 701–708; 10.1021/nn405364u (2014). 24308824

[b16] Juan M. L., Righini M. & Quidant R. Plasmon nano-optical tweezers. Nature Photonics 5, 349–356; 10.1038/Nphoton.2011.56 (2011).

[b17] Tian X., Fang Y. & Zhang B. Multipolar Fano Resonances and Fano-Assisted Optical Activity in Silver Nanorice Heterodimers. ACS Photonics 1, 1156–1164; 10.1021/ph5002457 (2014).

[b18] Hendry E. *et al.* Ultrasensitive detection and characterization of biomolecules using superchiral fields. Nat Nanotechnol 5, 783–787; 10.1038/Nnano.2010.209 (2010). 21037572

[b19] Liu N. & Pucci A. Plasmonic Biosensors Know Your Molecules. Nat Mater 11, 9–10; 10.1038/Nmat3199 (2012). 22169908

[b20] Mayer K. M. & Hafner J. H. Localized Surface Plasmon Resonance Sensors. Chem Rev 111, 3828–3857; 10.1021/Cr100313v (2011). 21648956

[b21] Fang Y. & Sun M. Nanoplasmonic waveguides: towards applications in integrated nanophotonic circuits. Light: Science & Applications 4, e294; 10.1038/lsa.2015.67 (2015).

[b22] Wu D. Y. *et al.* Surface Catalytic Coupling Reaction of p-Mercaptoaniline Linking to Silver Nanostructures Responsible for Abnormal SERS Enhancement: A DFT Study. J Phys Chem C 113, 18212–18222; 10.1021/Jp9050929 (2009).

[b23] Fang Y., Li Y., Xu H. & Sun M. Ascertaining p,p ‘-Dimercaptoazobenzene Produced from p-Aminothiophenol by Selective Catalytic Coupling Reaction on Silver Nanoparticles. Langmuir 26, 7737–7746; 10.1021/la904479q (2010). 20455558

[b24] Song P. *et al.* Remote-Excitation Time-Dependent Surface Catalysis Reaction Using Plasmonic Waveguide on Sites of Single-Crystalline Crossed Nanowires. Plasmonics 8, 249–254; 10.1007/s11468-012-9382-0 (2013).

[b25] Sun M. T., Huang Y. Z., Xia L. X., Chen X. W. & Xu H. X. The pH-Controlled Plasmon-Assisted Surface Photocatalysis Reaction of 4-Aminothiophenol to p,p ‘-Dimercaptoazobenzene on Au, Ag, and Cu Colloids. J Phys Chem C 115, 9629–9636; 10.1021/jp201002v (2011).

[b26] Huang Y. Z., Fang Y. R., Yang Z. L. & Sun M. T. Can p,p ‘-Dimercaptoazobisbenzene Be Produced from p-Aminothiophenol by Surface Photochemistry Reaction in the Junctions of a Ag Nanoparticle-Molecule-Ag (or Au) Film? J Phys Chem C 114, 18263–18269; 10.1021/jp107305z (2010).

[b27] Halas N. J., Lal S., Chang W. S., Link S. & Nordlander P. Plasmons in Strongly Coupled Metallic Nanostructures. Chem Rev 111, 3913–3961; 10.1021/Cr200061k (2011). 21542636

[b28] Brolo A. G. Plasmonics for future biosensors. Nature Photonics 6, 709–713; 10.1038/nphoton.2012.266 (2012).

[b29] Wang X. *et al.* Probing the Location of Hot Spots by Surface-Enhanced Raman Spectroscopy: Toward Uniform Substrates. ACS nano 8, 528–536; 10.1021/Nn405073h (2014). 24328390

[b30] Fang Y. R. & Huang Y. Z. Electromagnetic field redistribution in hybridized plasmonic particle-film system. Appl Phys Lett 102, 153108; 10.1063/1.4802267 (2013).

[b31] Fang Y. R., Tian X. R. & Huang Y. Z. Electromagnetic field redistribution in coupled plasmonic nanoparticle dimer-dielectric substrate system. Chem. Phys. Lett. 619, 139–143; 10.1016/j.cplett.2014.11.059 (2015).

[b32] Mark W. K. *et al.* Nanoparticle-Mediated Coupling of Light into a Nanowire. Nano Lett Vol. 7, No. 8, 2346–2350; 10.1021/nl071001t (2007). 17629348

[b33] Surbhi Lai. *et al.* Noble Metal Nanowires: From Plasmon Waveguides to Passive and Active Devices. Accounts Chem Res. Vol. 45, No. 11, 1887–1895; 10.1021/ar300133j 23102053

