# Supplementary material for: Electromagnetic field redistribution induced selective plasmon driven surface catalysis in metal nanowire-film systems
Source: Sci Rep. 2015 Nov 25;5:17223. doi: 10.1038/srep17223 (PMC4658649; doi:10.1038/srep17223)
Supplement: Supplementary Information [file srep17223-s1.doc]

**Supplemental Information**

# Electromagnetic field redistribution induced selective plasmon driven surface catalysis in metal nanowire-film systems

Liang Pan^1^, Yingzhou Huang^1*^, Yanna Yang^1^, Wen Xiong^2^, Guo Chen^2^, Xun Su^2^, Hua Wei^2^, Shuxia Wang^2*^and Weijia Wen^3^

^1^ Soft Matter and Interdisciplinary Research Center, College of Physics, Chongqing University, Chongqing, 400044, P. R. China

^2^ Department of Applied Physics, College of Physics, Chongqing University, Chongqing, 400044, P.R. China

^3^ Department of Physics, The Hong Kong University of Science and Technology, Clear Water Bay, Kowloon, Hong Kong, China

Corresponding authors. Emails: [yzhuang@cqu.edu.cn](mailto:yzhuang@cqu.edu.cn) (Y. Huang), and [wangshuxia@cqu.edu.cn](mailto:wangshuxia@cqu.edu.cn) (S. Wang)

**SERS spectra of 4NBT adsorbed on Au films at different positions(point 1-6) of Ag nanowires perpendicular excited by 633nm laser.** As the SEM image inset in Figure S1, two Ag nanowires with a 5.6μm adjacent part were located on the 4NBT monolayer adsorbed on Au film, where the diameters and lengths of two nanowires were 143nm, 179nm, 11.6μm, 5.6μm, respectively. To demonstrate the reproducibility of our experiment results, the Raman signals were collected at different positions of this Ag nanowire dimer-Au film system, in which the collection positions were indicated by colored arrows. The data present a good supporting to the reproducibility in our work. Furthermore, the collected Raman signals exhibited much larger intensities that it was almost 11 times greater at 1336 cm-1 Raman peak and 14 time’s greater at 1589 cm-1 Raman peak.

**
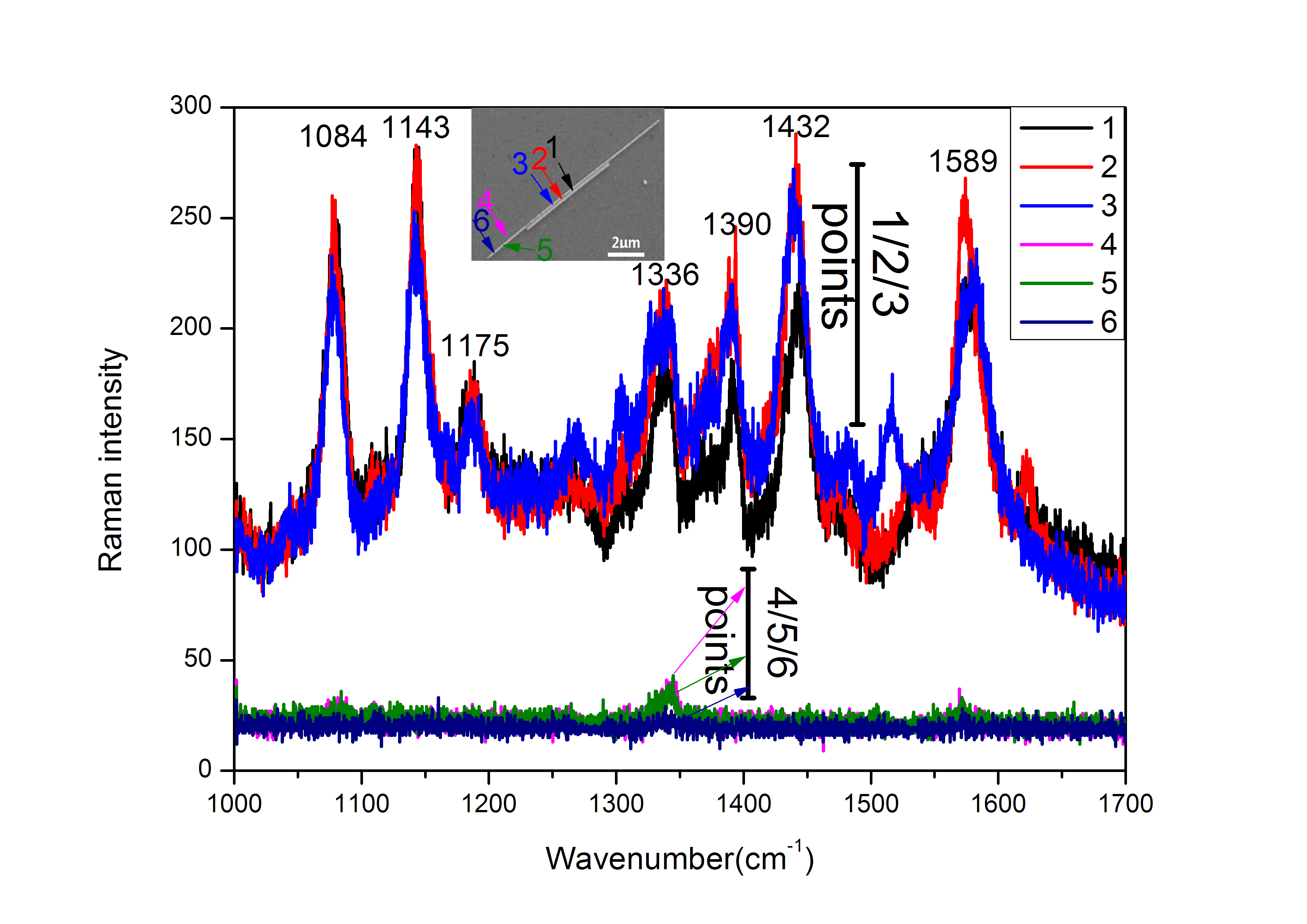
**

**Figure S1: SERS spectra of 4NBT adsorbed on Au film at different positions of Ag nanowires dimer.** Two Ag nanowires with a 5.6μm adjacent part were located on the 4NBT monolayer adsorbed on Au film, where the diameters and lengths of two nanowires were 143nm, 179nm , 11.6μm, 5.6μm, respectively.
